# BioGraph: unsupervised biomedical knowledge discovery via automated hypothesis generation

**DOI:** 10.1186/gb-2011-12-6-r57

**Published:** 2011-06-22

**Authors:** Anthony ML Liekens, Jeroen De Knijf, Walter Daelemans, Bart Goethals, Peter De Rijk, Jurgen Del-Favero

**Affiliations:** 1 Applied Molecular Genomics group, VIB Department of Molecular Genetics, Universiteit Antwerpen, Universiteitsplein 1, 2610 Wilrijk, Belgium; 2 Advanced Database Research and Modelling group, Department of Mathematics and Computer Science, Universiteit Antwerpen, Groenenborgerlaan 171, 2020 Antwerpen, Belgium; 3 Computational Linguistics and Psycholinguistics Research Center, Universiteit Antwerpen, Prinsstraat 13, 2000, Antwerpen, Belgium

## Abstract

We present BioGraph, a data integration and data mining platform for the exploration and discovery of biomedical information. The platform offers prioritizations of putative disease genes, supported by functional hypotheses. We show that BioGraph can retrospectively confirm recently discovered disease genes and identify potential susceptibility genes, outperforming existing technologies, without requiring prior domain knowledge. Additionally, BioGraph allows for generic biomedical applications beyond gene discovery. BioGraph is accessible at http://www.biograph.be.

## Rationale

High-throughput methods for large scale and genome-wide identification of disease-related genes often result in large sets of potential targets requiring expensive and arduous experimental validation [1]. For the high-throughput discovery of genes associated with disease (further referred to as 'disease genes'), it is necessary to identify functionally interesting research targets among large sets of candidates. The latter often requires a thorough understanding of possibly indirect functional relations between the research subject and its putative targets. However, one of the most common problems facing biomedical researchers today is finding or keeping up with the knowledge relevant to research interests in the shear amount of available literature and data. Especially when required information is functionally only indirectly connected to a researcher's main field of interest, the data deluge becomes unmanageable.

Based on the availability of large volumes of curated biomedical databases, various methods for gene prioritization have emerged in recent years [2]. These computational technologies rank putative disease genes with the goal of identifying true disease genes as prominent genes in the ranking. Computational technologies are complementary to conventional 'wet lab' gene discovery technologies in that they can support the prioritization and comprehension of, for example, associated regions from genome wide association studies or linkage studies, allowing researchers to more efficiently select the most compelling variants for further study. A common prioritization approach is the identification of potential causative genes that complement sets of known genes associated with disease, utilizing genetic interaction networks, regulatory networks or high-throughput datasets [3-5]. The statistical fusion of prioritizations from multiple, heterogeneous resources allows for ranking by incorporating diverse types of knowledge [6,7]. Alternatively, literature mining is a related research theme that employs natural language processing to extract biomedical information from the literature and to adopt this information for the discovery of new knowledge [8].

Prioritization platforms commonly lack an easily accessible user interface for the formulation of queries and the intelligible interpretation of the results. One common problem is that most of the data mining platforms are supervised, that is, they require prior domain knowledge from the user. For example, in disease gene prioritization techniques, it is commonly required to define a set of known disease genes on which the system can be trained for the identification of new genes. Since these training gene sets are subjective, they will consequently vary between users and outcomes are strongly dependent on them, and the robustness of the predictions becomes impaired. These platforms offer rankings of possible susceptibility genes, but often lack comprehensible support for these prioritizations. Often, rankings of research targets are offered without references to the literature, inhibiting the user from evaluating the rationale behind the predictions. Still, platforms that offer rationale and incentives for researching functional support are mostly limited to a specific domain of interactions. A common paradigm, for example, is to adopt protein or gene interaction networks for the construction of functional hypotheses, which excludes alternative functional explanations in support of the predictions. Here, we propose BioGraph, a user-friendly computational platform that strives to overcome such deficiencies by applying novel data mining techniques on integrated databases of diverse types of biomedical knowledge.

**Figure 1:**
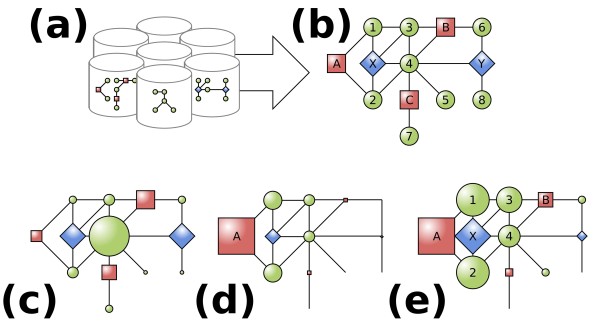
**Schematic representation of the data integration and data mining methodology**. **(a) **Public databases with heterogeneous biomedical relations are integrated into a common network. **(b) **Illustratively, genes (green circles), diseases (red boxes) and protein domains (blue diamonds) are related through gene-disease associations, gene-gene interactions and gene-domain annotations and integrated into a unified graph. **(c) **The *a priori *accessibility of each concept is computed by performing stochastic random walks to detect highly connected hubs in the network (area of a node scales with its rank score). **(d) **The *a posteriori *rank of each concept with respect to a source concept, in this case disease A, is computed by performing random walks with restarts in the source. **(e) **The posterior probabilities are adjusted using the prior probabilities to score the importance of each concept, specific to the source target (area of node scales with log of rank score). Genes (green circles) are ranked according to this score, gene 1 being most specific to disease A and gene 8 least specific.

Summarized, BioGraph provides an online resource and data mining method for the automated inference of functional hypotheses between biomedical entities. Assessment of these hypotheses can consequently be used for the ranking of targets in the context of a research domain, such as a disease. BioGraph's resource is a knowledge base that integrates many biomedical databases into a common network of heterogeneous relations. These databases are selected based on their practices of manual curation by experts, guaranteeing that the integrated knowledge is accurate and valid. Our methodology generates a map of relations linking biomedical research subjects to potential targets, such as diseases, genes, ontology annotations, pathways, and so on, and offers literature support for these putative functional hypotheses. Assessment of these hypotheses' plausibility and specificity to source and targets allows for various applications in the identification of promising research targets. Here, we focus on the genome-wide identification of susceptibility genes for heritable disorders. The overall framework of BioGraph's methodology is schematically represented in Figure 1.

## Methods and principles

### Integration of heterogeneous knowledge sources

BioGraph is based on the data integration of 21 publicly available curated databases containing biomedical relations (Table 1; Additional materials and methods in Additional file 1) between heterogeneous biomedical entities such as genes, diseases, compounds, pathways, ontology terms, protein domains, disease and gene families, and microRNAs.

**Table 1:** Integrated databases

Database	Concept 1	Relation	Concept 2	Literature references	Number of relations
BioGRID [25]	Gene/protein	PPI	Gene/protein	Yes	29,566
CTD [26]	Compound	Association	Gene/protein	Yes	62,336
	Compound	Association	Disease	Yes	5,438
	Gene/protein	Association	Disease	Yes	8,123
DIP [27]	Gene/protein	PPI	Gene/protein	Yes	1,524
GOA [28]	Gene/protein	Annotation	Gene Ontology term	No	26,949
HPRD [29]	Gene/protein	PPI	Gene/protein	Yes	149,036
IntAct [30]	Gene/protein	PPI	Gene/protein	Yes	37,258
InterPro [31]	Gene/protein	Contains	Protein domain/repeat/region	No	26,652
	Gene/protein	Is member of	Gene family	No	22,988
	Gene/gene family/protein domain/repeat/region	Annotation	Gene Ontology term	No	18,446
KEGG [32]	Gene/protein	Is part of	Pathway	No	14,100
	Gene/protein	Has metabolite	Compound	No	19,073
MeSH [33]	Disease	Belongs to	Disease (family)	No	21,282
MINT [34]	Gene/protein	PPI	Gene/protein	Yes	11,389
miR2Disease [35]	MicroRNA	Targets	Gene	Yes	2,615
	MicroRNA	Association	Disease	Yes	344
NetworKIN [36]	Gene/protein	Phosphorylates	Gene/protein	No	2,811
OMIM Morbid Map [11]	Gene/protein	Association	Disease	Yes	6,199
OMIM [11]	Disease	Is related to	Disease	Yes	2,467
TarBase [37]	MicroRNA	Targets	Gene	No	858

Overview of the 21 publicly available curated databases used to create BioGraph's heterogeneous knowledge base. Specific concept types were extracted from the various databases and integrated into a central graph. Note that these represent relations selected for *Homo sapiens *only. OMIM's disease-disease relations have been added after the data freeze of March 2010. CTD, Comparative Toxicogenomics Database; DIP, Database of Interacting Proteins; GOA, Gene Ontology Annotations; HPRD, Human Protein Reference Database; KEGG, Kyoto Encyclopedia of Genes and Genomes; MeSH, Medical Subject Headings; MINT, Molecular Interactions Database; OMIM, Online Mendelian Inheritance in Man; PPI, protein-protein interaction.

The integrated databases were selected based on their quality of relations with respect to curation methods and peer-reviewed references to the literature. Curated database producers employ domain experts to read and extract proven knowledge from the peer-reviewed scientific literature. Such processes of indexing, albeit time-consuming, ensure that the collected knowledge is accurate and complete, allowing for the successive establishment of new relations, for example, with BioGraph or related prioritization algorithms. We did not integrate databases constructed from high-throughput experiments with statistical or computational inferences where no manual curation of the indexed relations was performed. Such databases may include information of lower quality and consequently impair the predictive quality of consecutive data mining. We provide an assessment of each database's quality in the Results section.

The integrated databases in BioGraph consist of three types. (1) Curated databases (for example, Online Mendelian Inheritance in Man (OMIM) and various protein-protein interaction databases) constructed by manual extraction of published, peer-reviewed information about a specific type of information, guaranteeing the quality of the relations in these databases. (2) Curated ontology databases (for example, Gene Ontology (GO) and Medical Subject Headings) using hierarchical classifications of subjects. (3) Curated annotation databases (for example, GO Annotations (GOA) and Kyoto Encyclopedia of Genes and Genomes (KEGG) pathway database) that relate biomedical entities or concepts to ontology terms.

With regard to the integration of diverse databases with diverse identifiers for the concepts, each concept is provided with a distinct accession number, based on the Unified Medical Language System (UMLS) [9], to guarantee each concept's uniqueness. It should be noted that some of the integrated concepts (especially microRNAs and pathways) are underrepresented in UMLS. In these cases, we have extended the index of UMLS identifiers by these concepts' originating identifiers (for example, by adopting miRBase and KEGG pathway accession numbers). Relations between concepts are extracted from the knowledge resources, represented in a common format, annotated with semantic relation types (denoting the meaning of the relations, for example, 'protein interaction' or 'disease drug') and references to supporting literature, as provided by the integrated databases. All relations in the network are equally weighed independent of their support in the databases or the literature. We have experimented with weighing relations differently, dependent on the quality of the resource database, semantic type or references in the literature, but have not noticed a significant effect of such weights on test benchmarks, as discussed later. To sanitize the resulting network for the subsequent data mining algorithms, disconnected concepts from the largest connected network are removed and dangling concepts (that is, concepts connected to only one other concept) are pruned. As a result, the integrated network comprises 54,567 biomedical entities representing unique biomedical concepts and 425,353 unique relations among these entities, supported by 244,258 references to 52,866 items from the biomedical literature. The integrated network is frequently updated with updates of its dependent resources and the list of integrated databases may be appended with additional resources.

### Prioritization principle

Provided with the integrated network, one can intuitively conjecture that nearby concepts in the integrated network are related. Indeed, since functionally related concepts are connected in the graph, we may assume that concepts that are close but only indirectly related in the network may also be functionally related in the real world. However, empirical analysis of the network shows that most of the concepts in the network are interconnected in only a few steps. This indicates that the network shows so-called small-world properties. Indeed, there is a considerable abundance of highly connected nodes. For example, interactions of proteins with water and ATP compounds or functional annotations such as the location of a protein in membranes or protein binding are prevalent. These unspecific hubs serve as ubiquitous connections mediating short path lengths between functionally unrelated concepts. This characteristic of the network prevents successful prioritization using simple shortest path methods. Still, our prioritization technique relies on the detection of nearby concepts in the network with respect to a source concept, but we correct the ranking of concepts for their global accessibility in the network.

We provide a short technical summary of the methods here, but refer the interested user to the full implementation details in the Additional materials and methods in Additional file 1. We utilize stochastic random walks (trajectories on the network that consist of taking successive steps from one entity to a random related entity) on the knowledge network to measure the *a priori *importance or accessibility of concepts in a graph. This technique determines the global centrality of concepts in our integrated network. For this purpose, we compute the limit distribution that yields the probability of visiting the concepts when performing an infinite random walk on the integrated network. Google's PageRank algorithm [10] adopts a similar link analysis algorithm to rank web pages by their relative importance. Network hubs (top ranked concepts with a high prior probability) are generic and unspecific target concepts in the network (Additional Table 1 in Additional file 1). These hubs indicate important concepts for diverse biomedical processes, but should be avoided when trying to find relevant and non-obvious links between seemingly unrelated concepts.

For computing the vicinity of targets to a source concept in similarity to the prior probabilities, we compute the limit distribution of a stochastic model of random walks with restarts in the source concept (with probability 0.25 at each step). As such, we compute the *a posteriori *accessibility of each concept from the source concept, measuring the probability of visiting each target concept from the source disease, pathway, and so on. Concepts are scored by their posterior probability, divided by the square root of their respective prior probabilities and ranked with respect to this resulting score. In practice, for a gene prioritization query, a user of the web application provides a 'research subject' (for example, a disease, but also a pathway, a GO annotation or a gene may represent a research subject) and a list of 'research targets' (for example, putative genes or compounds) that need to be ranked in relation to the research subject. Our algorithm then assesses and ranks the relations between the source concept and each of the target concepts as above. Since any type of concept can be provided as the subject or target of a prioritization, our method does not require prior domain knowledge from the user, that is, there is no need to define a gene set of known disease-causing genes for the identification of related genes, which results in a more reproducible and robust user experience.

### Automated generation of functional hypotheses

**Figure 2:**
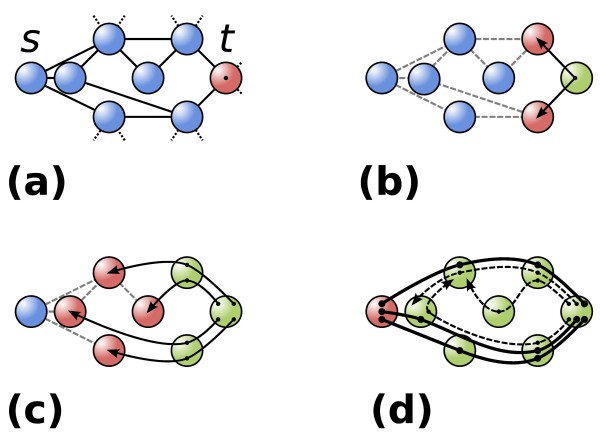
**Schematic representation of the backtracking heuristic to find most probable paths from a source concept *s *to a target *t***. **(a) **Assume a network with source and target concepts. For clarity, the nodes are ordered by their accessibility from *s *(leftmost nodes are most accessible, rightmost nodes least accessible). **(b) **As a first step in the backtracking process, we find the neighbors of the target *t*, leading in the direction of the source, that is, the neighbors of *t *with highest accessibility with respect to *s*. **(c) **The paths from the target are repeatedly expanded to include highly accessible nodes leading toward the source concept. Pruning of least probable paths keeps the growing set of paths to a workable size (not shown). **(d) **Most probable paths that arrive in the source (continuous lines) are considered as functional hypotheses linking the target to the source concept. Unfinished paths (dashed paths) continue being expanded until *k *paths between *s *and *t *have been found.

The method of performing random walks to determine the accessibility of target concepts implicitly generates ensembles of indirect paths between source and target concepts, which may serve as functional hypotheses for highly ranking targets. We can heuristically determine highly probable simple paths, that is, paths that do not contain cycles, of the random walk that starts in the source concept and ends in the target concept by adopting backtracking (Figure 2). The backtracking heuristic incrementally builds partial candidate paths, starting from the target to the source, while abandoning least likely paths along the way, leading to valid and specific paths that offer incentives for further functional research. A detailed description of the heuristic is available in the Additional materials and methods in Additional file 1.

The resulting set of paths is presented to the user as a network with putative hypotheses linking the source to the target. Each directed edge represents a supporting relation among intermediate concepts, with annotated semantic meanings and literature references intelligibly supporting the relation for evaluation by the user. In cases where the target is highly ranked, specific and relevant connections and concepts are included in the constructed hypotheses. If the functional hypotheses linking concepts is limited to visiting general hub concepts, this is usually a sign that the linked source and target concepts can be considered unrelated, reflected by a bad ranking score.

## Results

In order to assess the quality of BioGraph in prioritizing interesting research targets, we study its application in the identification of genes known to be associated with disease. Test sets of proven disease-related genes were selected from the OMIM Morbid Map database and Comparative Toxicogenomics Database (CTD). OMIM Morbid Map contains several thousand diseases and disease genes with a proven underlying molecular basis, manually selected and indexed from the peer-reviewed medical literature by experts [11]. Similar to the curation process for OMIM Morbid Map, the CTD employs professional biocurators who read and manually curate the literature to derive proven relations among genotypes and phenotypes, ensuring that the indexed data are valid and accurate.

We have adopted the BioGraph framework to prioritize all human genes in the context of diseases selected from these databases and evaluate the positions of the diseases' proven susceptibility genes in this ranking. We then compute sensitivity and specificity values and observe the area under the receiver operator characteristic (ROC) curve (AUC) as the standard performance measure for analyzing the quality of prioritizations or classifications [12]. A perfect ranking algorithm that manages to put the true disease genes at the top would score 100% on such a test, where random rankings score 50%. Provided with a reliable and valid AUC measure, it can be interpreted as the probability that when we randomly pick one positive and one negative example, the prioritization algorithm will assign a higher rank to the positive example than to the negative. An algorithm that scores well on this assessment is thus likely to identify disease-associated genes as high-ranking genes and vice versa.

### Disease-gene prioritization benchmark

As a first application, we analyzed the performance of our platform in prioritizing known disease genes among all genes in our integrated knowledge base. For testing a known disease-gene association, we first removed the link between the disease and its susceptibility gene from the knowledge base. We then ranked all genes in the network in relation to the disease and evaluated the ranking of the test gene. If the test genes ranked high, BioGraph allows retrieval of these genes as valid disease genes based on integrated information linking the disease to its susceptibility gene. For this test, we adopted published benchmarks and compared our prediction performance to that of Endeavour.

Endeavour is a related and mature technology for gene prioritization [6], which adopts a data fusion method to build statistical models of known disease-causing genes with respect to various data sets. Using order statistics, genes are prioritized by measuring the matching quality of test genes to these training profiles.

We have computed the performance of BioGraph's prioritization method for the disease-gene prioritization benchmark initially published to evaluate the performance of Endeavour. This benchmark consists of 627 genes known to cause 29 diseases, selected from the OMIM database, of which 609 disease genes are present in our integrated knowledge base [6].

Benchmarking a disease gene with BioGraph requires that each disease gene is evaluated by first removing the direct relations between the gene and the disease from the integrated network to ensure that the relation to be prioritized is not already in the network. Moreover, variants of the disease (for example, subtypes or syndromes that have the disease as one of its symptoms) are also disconnected from the disease gene. In order to identify these related diseases, we have selected the diseases for which at least one of its UMLS synonyms has the original disease's name as its substring. For example, we identify Charcot-Marie-Tooth disease, type 4C as a related disease to Charcot-Marie-Tooth or Alport's disease as related to Deafness, since a synonym of Alport's disease is Nephritis with nerve deafness. This method provides an objective interpretation of the benchmark by guaranteeing that no prior direct information can be exploited by our prioritization algorithm. Subsequently, for the prioritization of a disease gene, we perform a ranking of the integrated network's 16,912 known human genes with respect to the disease concept.

For benchmarking Endeavour, each disease-gene relation was tested by removing the gene from the disease's known gene set, by training Endeavour on the remaining disease genes and by ranking the gene among a set of 99 random test genes. For both platforms, we adopt the AUC for analyzing the quality of these prioritizations.

The mean AUC for BioGraph's prioritization of disease genes among all human genes is 92.92%, where the reported AUC for Endeavour in prioritizing disease genes among 99 random genes is 86.6% [6]. Additional Table 2 in Additional file 1 lists the AUC scores for the prioritization results per disease. Of the 609 disease genes in the benchmark, 181 prioritizations (29.72%) are ranked in the top 1% of the test set of all genes and 449 (73.73%) are ranked in the top 10%. In other words, in an experimental application where a causative gene is among a set of 99 random genes, BioGraph is consequently expected to rank the defecting gene as the top gene in 29.72% of the cases and in the top 10 with probability 73.73%.

**Table 2:** Top inferred genes for schizophrenia

Number	Gene	Prioritization hypothesis	SZ association studies
1	*PRL*	Affected by the antipsychotics aripiprazole and risperidone, neuroactive ligand-receptor interaction, associated with autistic disorder	No association studies. Associated with autistic disorder [16]
2	*ARID4B*	Target of mir-20b	No association studies
3	*HTR1A*	Related to *HTR2A*	Positive association [19]
4	*DRD2*	Related to *DRD3*	Positive association [20]
5	*DNMT3B*	Target of mir-29*, related to *COMT*, folic acid	Positive association [21]
6	*DNMT3A*	Target of mir-29*, related to *COMT*, folic acid	No association studies
7	*FSTL1*	Target of mir-206	No association studies
8	*SYN3*	Related to *SYN2*	No association found [23]
9	*MYLIP*	Target of mir-20b, involved in CNS development	No association studies
10	*EFEMP2*	Target of mir-346	No association studies
11	*UTRN*	Interacts with *DISC1*, target of mir-206	No association studies
12	*OMG*	Myelin sheet, interacts with *RTN4R*, axonogenesis	Weak positive association [22]. Putatively associated with mental retardation [38]
13	*BACE1*	Target of mir-29*, Alzheimer's disease	No association studies. Schizophrenia-like phenotypes in *BACE1*-null mice [39]
14	*HIPK3*	Target of mir-20b	No association studies
15	*TAC1*	Target of mir-206, axonal and synaptic transmission	No association studies. Down-regulated in psychosis [40]
16	*ATXN1*	Interacts with *ZNF804A *and *AKT1*	Positive association [18]
17	*SYN1*	Related to *SYN2*	No association studies. Associated with epilepsy [41]
18	*RTN4IP1*	Interacts with *RTN4R*, neurite growth	No association studies
19	*CDKN1A*	Interacts with *AKT1*, target of mir-20b	No association studies
20	*LINGO1*	Interacts with *RTN4R*, axonogenesis, CNS development	No association studies. Associated with essential tremor and Parkinson's disease [42]

BioGraph top inferred genes for schizophrenia that are not known as direct relations in the integrated network. Prioritizations are based on a data freeze of September 2009 to retrospectively verify predictions in more recent literature. CNS, central nervous system.

The benchmark indicates that our prioritization approach yields a considerable improvement over mature technologies. There are two noteworthy differences in the experimental benchmarking design. Our platform does not require a training set of known disease-causing genes since it will implicitly base prioritizations on integrated disease-gene associations in addition to other heterogeneous types of integrated knowledge of the disease. This has a major advantage for the user since no prior knowledge of the disease is required. Secondly, our platform provides a ranking of the disease gene in relation to all known genes, where Endeavour ranks disease genes among a random set of 99 non-disease genes.

As a quality control of the integrated databases, we have assessed the effect of each database on the benchmarking results by leaving out one database at a time and by assessing the prioritization algorithm on the Endeavour benchmark. This experiment showed that none of the included databases significantly harms the overall prediction capabilities. Conversely, it should be noted that some databases (most specifically CTD gene-disease, GOA and Medical Subject Headings) are essential for successful prioritization, since leaving out these databases has a significantly negative impact on the benchmarks. More information on these quality checks is available in the Additional materials and methods in Additional file 1.

### Ranking recently discovered disease genes

In the above benchmark tests, well-known disease genes are expected to rank high. Indeed, important susceptibility genes usually become the subject of intensive research efforts. Consequently, a literature and database bias may exist toward indirect evidence linking a gene to a disease in the integrated databases. Since BioGraph is capable of using this indirect evidence, the literature bias of important disease genes may strengthen the predictive power of our algorithm. To remove this bias, we can more objectively evaluate the platform by ranking recently discovered disease-gene relations that are not present in the knowledge base.

Provided with the integrated network for which the resource datasets were frozen in March 2010, we identified all recently curated additions of human disease-gene relations from the July 2010 releases of the OMIM Morbid Map (15 new disease genes) and CTD (830 direct, non-inferred relations) that are not present as direct relations in the knowledge base from March 2010. This yielded 845 recent disease-gene relations for which the ranks in the disease's genome-wide prioritization have been determined based on the integrated network of March 2010.

**Figure 3:**
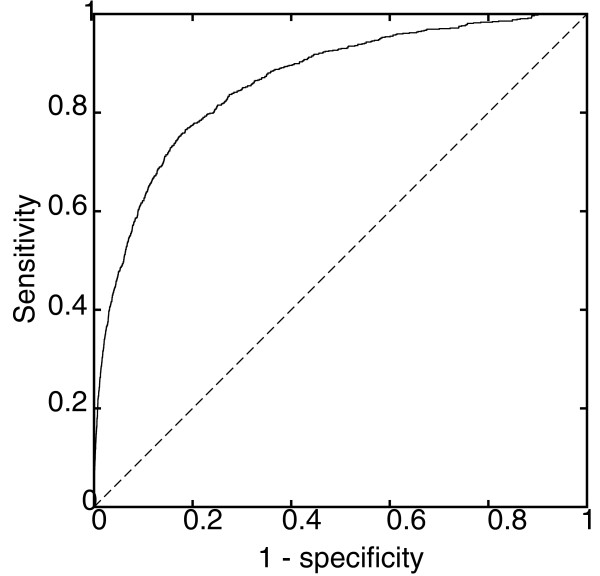
**ROC curve of prioritization performance on 845 recent disease-gene relations**. The performance of BioGraph prioritizations is 86.14%, confirming the relations recently added to the resource databases but not present in the integrated database. The diagonal dashed line represents a theoretical random algorithm.

Figure 3 shows the ROC curve of the combined results, with AUC 86.14%. Of the 845 curated disease genes, 189 prioritizations (22.73%) are ranked in the top 1% of the test set consisting of all genes for its corresponding disease and 524 (62.01%) are ranked in the top 10%. The median rank of a disease gene is in the top 6.04%.

## Applications

The above benchmarks demonstrate that BioGraph is capable of retrospectively finding or confirming existing disease genes, indicating that we can adopt the method to predict putative susceptibility genes for heritable diseases. Feasible applications of the framework are the identification of functionally interesting genes from sets of candidate genes - for example, in the identification of promising genes in linked regions, copy number variation regions or for the identification of genes through genome-wide association or expression studies.

Additionally, the automated construction of hypotheses is of interest to explore genetic/genomic findings in peer-reviewed functional support. Collecting functional support for newly discovered disease-gene associations is not always obvious, especially when the functional evidence is indirect and spans several fields of interest. With the advent of high-throughput methodologies and torrents of published material to substantiate these findings, detecting relevant information has become a laborious process where computational techniques, such as those presented here, allow for these processes to be automated.

Beyond applications in genetics and genomics, the framework can similarly be adopted to prioritize or to determine functional support for biomedical relations other than disease-gene associations - for example, in linking drug compounds, annotation terms, pathways, and so on - making the framework a very versatile tool in the discovery of diverse types of biomedical knowledge. In one feasible application, BioGraph can be adopted to determine functional interactions between drug compounds and for the *in silico *exploration of drug-drug interactions or the prioritization of identifying compounds in screening pipelines. Another example application is the computational inference of clinical biomarkers related to pathways, biochemical functions or disease processes, building on the various integrated types of concepts, relations and integrated literature references to detect promising candidates.

### Genome-wide prioritization of genes related to schizophrenia

To illustrate possible applications of the framework, we have employed the platform to predict candidate genes for schizophrenia (SZ) and substantiate the top predictions with support adopting the automatically generated functional hypotheses.

SZ is a common neuropsychiatric genetic disorder with approximately 1% prevalence and with 64% heritability. It is characterized by a constellation of symptoms, including hallucinations and delusions, and symptoms such as severely inappropriate emotional responses, disordered thinking and concentration, erratic behavior, as well as social and occupational deterioration [13,14].

The newly identified genes are indirectly inferred from the integrated knowledge, but not directly associated in our gene-disease resource databases. The predictions in this section are based on a dataset freeze of the integrated databases from September 2009. This data freeze allows us to test if the predicted genes have been observed in genetic studies since the data freeze. Table 2 shows the top 20 inferred BioGraph genes with respect to the SZ concept, as designated by its UMLS accession ID [UMLS:C0036341], and a short summary of the hypotheses of their relatedness with SZ by our platform.

*PRL*, the top inferred gene that is not a known disease-causing gene for SZ, encodes the prolactin hormone, of which the most commonly known function is to stimulate lactogenesis. Prolactin's relation to SZ is important, especially due to the effects of dopamine-regulating drugs aripiprazole and risperidone on the expression of prolactin and their adverse hyperprolactinemia-associated side effects [15] where the secretion of prolactin is regulated by dopamine, following the current dopamine hypothesis of SZ. We did not find published association studies of prolactin with SZ, although an association with autistic spectrum disorder was reported [16]. Additionally, *PRL *is located on chromosome 6p22.3, which is linked to SZ through *DTNBP1 *[17] and *ATXN1 *[18]. Although no causal associations have been shown between prolactin function and SZ, BioGraph hypothesizes *PRL *as a likely candidate gene for SZ.

**Figure 4:**
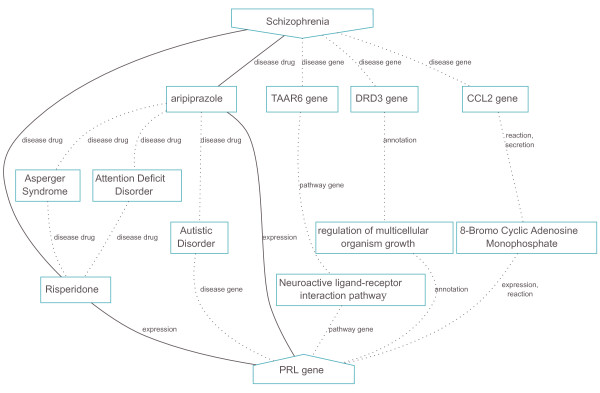
**Schematic representation of the top ten automatically generated hypotheses supporting the susceptibility of *PRL *in relation to schizophrenia**. Solid, dashed and dotted line styles represent the importance of the link in descending order, that is, the probability to visit the relation to reach the target gene concepts while performing random walks from the source schizophrenia concept. All links are grounded in their originating integrated curated knowledge bases, annotated with their semantic meanings and enriched by their references to the literature (not shown).

The automatically inferred hypotheses by BioGraph that support the high ranking of *PRL *for SZ are along the lines of current understandings and are schematically shown in Figure 4. The most likely indirect links between SZ and *PRL *are through the antipsychotic compounds aripiprazole and respiredone, which are both dopamine antagonists affecting the expression of prolactin. This hypothesis also shows that both compounds are adopted as drugs for attention deficit disorder, Asperger syndrome and autistic disorders. Additionally, *PRL *is associated with autistic disorder, strengthening the importance of it for psychiatric disorders. Additional paths from SZ visit SZ-associated genes and commonalities among these genes with *PRL*; *TAAR6 *and *PRL *are both genes in the neuroactive ligand-receptor interaction pathway; *CCL2 *and *PRL *are both regulated by 8-bromo cAMP, a derivative of cyclic AMP; *DRD3 *and *PRL *share the GO annotation 'Regulation of multicellular organism growth'. These relations may serve as indicators for determining the putative functional involvement of *PRL *in the etiology of SZ.

**Figure 5:**
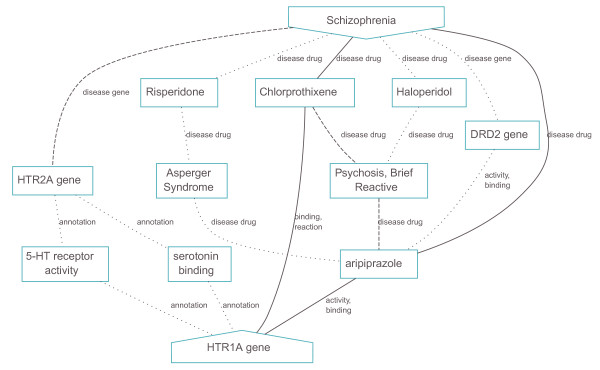
**Schematic representation of the top ten automatically generated hypotheses supporting the susceptibility of *HTR1A *in relation to schizophrenia**.

Figure 5 provides hypothetical evidence for the involvement of the second inferred candidate gene *HTR1A *(serotonin receptor 1A) with SZ. The main hypothesis is driven by the receptor's interaction with the antipsychotic drugs aripiprazole and chlorprothixene. *HTR1A *is additionally linked to its paralog *HTR2A*, a known susceptibility gene for schizophrenia, via GO annotations on serotonin binding activity. Although our integrated disease-gene databases (OMIM Morbid Map and CTD) have not indexed *HTR1A *as a schizophrenia susceptibility gene, variants in the gene have previously been shown to be associated with schizophrenia and other psychopathologies [19]. This example shows that BioGraph is capable of identifying known disease genes, even if these gene-disease associations are not in the integrated resources.

Significant associations with SZ and polymorphisms in 4 of the top 20 ranked genes, namely *HTR1A *[19], *DRD2 *[20], *DNMT3B *[21] and *ATXN1 *[18], have previously been shown. These known disease-gene relations are not indexed by our integrated databases, but were successfully prioritized by our data mining platform. Most notably, significant association of polymorphisms in *DNMT3B *with SZ was only reported in October 2009, where the data for our predictions are based on integrated databases from September 2009, demonstrating the usefulness of the currently proposed prioritization technique. Additionally, the highly ranking *OMG *gene has been shown to be associated with SZ, warranting replication studies for confirmation [22]. *SYN3 *is the only gene in the top 20 for which several association studies have been performed, but where the findings show no support for *SYN3 *as a SZ susceptibility gene [23].

For the remaining 15 of the top 20 genes, we did not find published association studies to support or contradict possible roles of these genes in SZ, although for some genes associations with SZ-like symptoms or related psychiatric and various neurological disorders have been shown, supporting the putative role of these genes in SZ (Table 2).

## Discussion

We have constructed BioGraph, an integrated network of curated relations from heterogeneous knowledge sources, such as disease-gene-compound associations, protein-protein interactions, GO and pathway annotations, microRNA targets, protein domains, and so on. In order to guarantee the accurateness of the integrated knowledge, the integrated databases were selected based on their curation processes for the indexing of knowledge from the peer-reviewed scientific literature. We show that the automated generation of functional hypotheses in this integrated network of biomedical knowledge allows the successful prioritization and identification of research targets in the context of a research subject. More specifically, we can successfully identify proven disease genes for hereditary diseases as highly ranking genes among all human genes in the context of their disease and vice versa. We have shown that ensembles of highly probable walks through this network can be adopted to successfully rank putative relations among non-obvious and indirectly associated concepts, with a focus on adopting these automatically generated hypotheses for the prioritization of possible susceptibility genes of diseases. The prioritization and automated hypothesis generation platform is available as a web service [24].

BioGraph offers a range of significant improvements over leading prioritization platforms for *in silico *identification of disease-related genes. Most notably, and in contrast with other methods, our approach is unsupervised and does not require prior domain knowledge from the user. This removes possible user biases and problems with prediction robustness in common supervised machine learning approaches that require, for example, training sets of known disease causing genes to define the subject of an analysis. Furthermore, highly ranked targets are grounded in comprehensible functional hypotheses, consisting of refereed relation paths in support of the prioritization. Since our method is based on the integration of heterogeneous knowledge sources, the generated hypotheses offer richer semantics about inferred biomedical relations compared to related data mining efforts in, for example, gene and protein interaction networks.

Tests on published benchmarks (AUC 92.92%) show that our prioritization method outperforms leading technologies and notable differences in the rankings are supported by comprehensible hypotheses that confidently support the prioritization. In experimental cases where an accountable gene needs to be identified in a set of 100 genes, BioGraph prioritizes the gene among the top 10 genes in 73.73% of the cases. We showed that BioGraph is able to retrospectively confirm recent disease-gene associations to the integrated databases (AUC 86.14%). Additionally, relations that have been confirmed in recent publications were successfully predicted. For example, BioGraph ranked *DNMT3B *as a top ranking SZ susceptibility gene using integrated data frozen in September 2009 while this association was published in October 2009. Additionally, of the top 20 prioritized inferred genes for schizophrenia, 4 disease genes were not indexed by the integrated resources but are confirmed as true associations by the literature.

Finally, we would like to note that, although the focus of the applications of BioGraph in this paper is in the ranking of disease-gene relations, the presented methodology is generic and applicable in various biological research settings requiring the construction of intelligent and intelligible hypotheses among interrogated concepts. One may use the platform, for example, to identify diseases related to a pathway of interest, or to enrich *a priori *defined gene sets to determine related ontology terms, compounds or protein domains.

## Abbreviations

AUC: area under the ROC curve; CTD: Comparative Toxicogenomics Database; GO: Gene Ontology; GOA: Gene Ontology Annotations; KEGG: Kyoto Encyclopedia of Genes and Genomes; OMIM: Online Mendelian Inheritance in Man; ROC: receiver operator characteristic; SZ: schizophrenia; UMLS: unified medical language system.

## Competing interests

The authors declare that they have no competing interests.

## Authors' contributions

WD, PDR, JDF and BG conceived the project. AL, JDK and PDR created the BioGraph resource, data miner and hypothesis generator, designed and carried out the performance tests and built the web service. All authors have read and approved the manuscript for publication.

## Supplementary Material

Additional file 1 **Additional materials and methods and Additional Tables**. Detailed methods describing technicalities of the database integration and algorithms, with the following sections. Knowledge integration: detecting hub nodes by computing *a priori *probabilities with random walks; computing *a posteriori *probabilities and ranking relations; backtracking heuristic for the automated generation of functional hypotheses; additional results. Additional Table 1: top 50 hubs or highest ranking concepts of the computation of the *a priori *rank score in the integrated network. Additional Table 2: area under the receiver operator characteristic (ROC) curve (AUC) for the prioritization of disease genes in the Endeavour benchmark. Additional Table 3: effect on the Endeavour benchmark after leaving out each separate database from the data integration process. Click here for file
