# Supplementary material for: BioGraph: unsupervised biomedical knowledge discovery via automated hypothesis generation
Source: Genome Biol. 2011 Jun 22;12(6):R57. doi: 10.1186/gb-2011-12-6-r57 (PMC3218845; doi:10.1186/gb-2011-12-6-r57)
Supplement: Additional file 1 — Additional materials and methods and Additional Tables. Detailed methods describing technicalities of the database integration and algorithms, with the following sections. Knowledge integration: detecting hub nodes by computing a priori probabilities with random walks; computing a posteriori probabilities and ranking relations; backtracking heuristic for the automated generation of functional hypotheses; additional results. Additional Table 1: top 50 hubs or highest ranking concepts of the computation of the a priori rank score in the integrated network. Additional Table 2: area under the receiver operator characteristic (ROC) curve (AUC) for the prioritization of disease genes in the Endeavour benchmark. Additional Table 3: effect on the Endeavour benchmark after leaving out each separate database from the data integration process. [file gb-2011-12-6-r57-S1.DOC]

1. BioGraph: Unsupervised Biomedical Knowledge Discovery via Automated Hypothesis Generation
2. Authors: Anthony M.L. Liekens^1,*^, Jeroen De Knijf^2^, Walter Daelemans^3^, Bart Goethals^2^, Peter De Rijk^1^, Jurgen Del-Favero^1^
3. ^1^Applied Molecular Genomics group, VIB Department of Molecular Genetics, Universiteit Antwerpen, Universiteitsplein 1, 2610 Wilrijk, Belgium
4. ^2^Advanced Database Research and Modelling group, Department of Mathematics and Computer Science, Universiteit Antwerpen, Groenenborgerlaan 171, 2020 Antwerpen, Belgium
5. ^3^Computational Linguistics and Psycholinguistics Research Center, Universiteit Antwerpen, Prinsstraat 13, 2000, Antwerpen, Belgium
6. *Corresponding author: Anthony Liekens, Applied Molecular Genomics group, VIB Department of Molecular Genetics, Universiteit Antwerpen, Universiteitsplein 1, 2610 Wilrijk, Belgium, [anthony@liekens.net](mailto:anthony@liekens.net)
   - 1. Additional Materials and Methods
   1. Knowledge integration

Biomedical databases contain *relations* of heterogeneous semantic meanings (interaction, association, annotation, hierarchical organization, etc.) among diverse biomedical *concepts* (such as genes and proteins, diseases, compounds, pathways, Gene Ontology^[43]^ terms, protein domains and microRNAs). We have integrated a large set of such resources (BioGRID^[25]^, CTD^[26]^, DIP^[27]^, GOA^[28]^, IntAct^[27]^, InterPro^[31]^, KEGG^[32]^, MeSH^[33]^, MINT^[34]^, miR2Disease^[35]^, NetworKIN^[36]^, OMIM^[11]^, TarBase^[37]^ and UniProtKB^[44]^) describing binary biomedical associations between specific biochemical concepts in *Homo sapiens* into a common relational network.

The databases' constituent concepts – represented by nodes in the integrated network – were mapped to unique UMLS^[9]^ identifiers, where possible. Since microRNAs, protein domains and pathways are underrepresented by the UMLS ontology, their original accession identifiers (from miRBase^[45]^, InterPro and KEGG, respectively) were retained. By adopting the UMLS ontology and its *metathesaurus* to centralize the identification of the various biomedical concepts, the resulting network – where edges are represented by interactions – becomes relational, in that no concept occurs more than once in the graph. By avoiding this redundancy upon the construction of the relational network, successive data mining of the integrated resources, as discussed later on, allows for the discovery of undeviating routes. Additionally, the centralization with UMLS entities allows for the flexible incorporation of additional concept types beyond the incorporated types and their inherent interactions.

Consequently, as a first step in the integration, we have created indices for the translation of concept from diverse sources to UMLS IDs, uniquely representing each concept, as follows[[1]](#footnote-2):

- HUGO gene name nomenclature entries and their corresponding HGNC IDs are present in the UMLS Metathesaurus, and can directly be translated to UMLS IDs
- MeSH concept IDs are present in the UMLS Metathesaurus, and can directly be translated to UMLS IDs
- OMIM disease concepts and genes are present in the UMLS Metathesaurus, and can directly be translated to UMLS IDs
- UniprotKB proteins are annotated by the HUGO names of their originating genes, which can be translated to UMLS IDs as above
- RefSeq^[46]^ sequences are annotated by UniprotKB IDs, which can be translated to UMLS IDs as above
- Gene Ontology^[43]^ term IDs are present in the UMLS Metathesaurus, and can directly be translated to UMLS IDs
- InterPro protein domains, repeats and other region types that are adopted for the annotation of genes are not present in UMLS. Our list of UMLS concepts has consequently been extended by the InterPro domain concepts to allow for these annotations, using their proper IPR* identifiers.
- KEGG pathways are only partly present in UMLS. The list of UMLS concepts has been extended by KEGG pathway concepts to allow for gene-pathway annotations, using their proper KEGG:hsa* identifiers
- KEGG gene identifiers are annotated with their HUGO names, which can directly be translated to UMLS IDs
- KEGG enzyme identifiers are annotated with their Enzyme Commission identifiers, which are linked with their corresponding genes represented as HUGO names, which can directly be translated to UMLS IDs. Note that in the case of a KEGG enzyme, multiple genes may be inferred from this translation
- KEGG compound identifiers are annotated with their Chemical Abstracts Service (CAS) registry numbers, which are present in the UMLS Metathesaurus and can be translated to UMLS IDs
- Disease Ontology^[47]^ disease identifiers are present in the UMLS Metathesaurus, and can directly be translated to UMLS IDs
- The set of miRNA identifiers in the UMLS Metathesaurus is incomplete and the thesaurus does not allow miRBase^[45]^ identifiers to be translated to UMLS identifiers. All microRNAs in the network are annotated with their miRBase identifiers and supplemented to the existing concepts

Edges of the integrated relation network describe associations among the concept nodes in the graph. The relations of the integrated databases provide different types of interactions, each with specific semantic annotations. The integrated databases were chosen with respect to their ability to describe elementary relationships that may yield chains of functional associations among more distant concepts upon integration and consecutive analysis. BioGRID, DIP, IntAct and MINT databases supply protein-protein interactions; gene-disease relations are extracted from OMIM and CTD; CTD also provides associations among compounds on one hand, and genes and diseases on the other; GOA and InterPro provide us with Gene Ontology annotations for genes and protein domains; InterPro also provides protein domain and gene family annotations for proteins; pathway memberships for proteins, and enzyme-metabolite relations have been extracted from KEGG; hierarchical organization of diseases/compounds and families of diseases/compounds have been integrated from MeSH; NetworKIN provides kinase-substrate relations; TarBase and miR2Disease contains curated targets of microRNAs, where miR2Disease further contains miRNA-disease associations. Additional databases with relationships capable of providing functional relationships can effortlessly be integrated to extend the current network.

Extracting semantics from the integrated databases implies a different approach for each database.

1. Most databases (e.g., protein-protein interaction databases or gene ontology annotations) only index one type of relation and do not offer any further clues on subtypes of this relation (for example, types of PPIs, such as localization, binding, phosphorylation, … are missing), so all relations extracted from these are annotated with the same relation type (e.g., “protein interaction” or “annotation,” respectively).
2. Some databases, however, provide specificities of the relations in a limited ontology (e.g., CTD’s chemical-gene database provides types of interactions, e.g., “phosphorylation,” “reaction,” “export,” “binding,” “expression,” …). Integrated relations were annotated with their respective meanings in a common ontology that spans all databases (e.g., “phosphorylation” is used for all phosphorylation relations, independent from their origin database)

The following list details the integration steps for each of the databases. If an integrated concept needs to be translated to its UMLS ID, but UMLS (or its Additional concepts from InterPro, KEGG or miRbase) does not contain an instance of this concept, the relation among the unknown concepts is ignored.

- BioGRID protein-protein interactions: Provided in TAB format from BIOGRID-ORGANISM-Homo_sapiens-2.0.62.tab.txt from [49]. Interacting proteins are provided by their HUGO gene names, which can directly be translated to UMLS accessions. Semantic annotations: *protein interaction*, *genetic interaction*
- CTD (The Comparative Toxicogenomics Database) gene-compound relations: Source [50]. Compounds are provided by their MeSH ID, genes by their HUGO names, which can directly be translated to UMLS accessions. Semantic annotations: *abundance, acetylation, activity, acylation, alkylation, amination, binding, carbamoylation, carboxylation, chemical synthesis, cleavage, cotreatment, degradation, ethylation, export, expression, farnesylation, folding, geranoylation, glucuronidation, glutathionylation, glycation, glycosylation, hydrolysis,hydroxylation, import, localization, metabolic processing, methylation, mutagenesis, N-linked glycosylation, nitrosation, oxidation, phosphorylation, prenylation, reaction, reduction, response to chemical, ribosylation, secretion, splicing, stability, sulfation, sumoylation, transport, ubiquitination, uptake*
- CTD disease-compound relations: Source [51]. Compounds are provided by their MeSH ID, diseases by their OMIM ID, which can directly be translated to UMLS accessions. Relations are annotated with PubMed literature references. Semantic annotations: *disease drug*, *disease marker*
- CTD gene-disease relations: Source [52]. Genes are provided by theiur OMIM ID, genes by their HUGO names, which can directly be translated to UMLS accessions. Relations are annotated with PubMed or OMIM literature references. Semantic annotations: *disease gene*
- DIP protein-protein interactions: Source [53]. Interacting proteins are provided as UniprotKB or RefSeq IDs, which can be translated to UMLS accessions. Relations are annotated with PubMed literature references. Semantic annotations: *protein interaction*
- GOA gene ontology annotations: Source [54]. Genes are provided as their HUGO gene names and ontology annotations are provided as their Gene Ontology IDs, which can directly be translated to UMLS accessions. Relations are not annotated with literature references. Semantic annotations: *annotation*
- HPRD protein-protein interactions: Source [55]. Interacting proteins are annotated with their HUGO gene names, which can directly be translated to UMLS accessions. Relations are annotated with PubMed literature references. Semantic annotations: *protein interaction*
- IntAct protein-protein interactions: Source [56]. Interacting proteins are annotated with their UniProtKB accessions, which can be translated to UMLS accessions. Relations are annotated with PubMed literature references. Semantic annotations: *protein interaction*
- InterPro gene-domain associations: Source [57]. Genes are annotated with their HUGO names, which can directly be translated to UMLS accessions. Protein domains are annotated by their InterPro accessions denoting protein domains, repeats and other regions, which are not available in UMLS. The accession numbers from InterPro have been supplemented to the list of UMLS accessions to allow for the inclusion of these annotations in the integrated network. Relations are not annotated with literature references. Semantic annotations: *annotation*
- InterPro gene-gene family annotations: Source [58]. Genes and gene families are provided as InterPro accession identifiers, which are not available in UMLS and have been incorporated in the concept accession list. Relations are not annotated with literature references. Semantic annotations: *ontology*
- InterPro gene-GO term annotations: Source [58]. InterPro concepts are provided by their InterPro accession identifiers, which are not available in UMLS and have been incorporated in the concept accession list. Gene ontology annotations for these domains are provided by their Gene Ontology identifier, which can directly be translated to UMLS identifiers. Relations are not annotated with literature references. Semantic annotations: *disease gene*
- KEGG gene-pathway associations: Source [59]. Genes are provided by their HUGO names, which can directly be translated to UMLS accessions. Pathways of which these genes are part, can either be part of UMLS, or not. In the case that a KEGG pathway identifier is indeed present in UMLS’ concepts, the UMLS Metathesaurus allows for the direct translation of a KEGG identifier to a UMLS accession number. In the other cases, the pathways are missing from UMLS but have been supplemented to the list of concepts. Relations are not annotated with literature references. Semantic annotations: *pathway gene*
- KEGG gene-compound associations: Source [60]. Contrasting with the other integrations of knowledge databases, KEGG’s associations between genes and their proteins’ metabolites have not been downloaded, but were extracted from the KEGG database by accessing its web services. As our initial resource, we adopt KEGG’s enzyme database (ftp://ftp.genome.jp/pub/kegg/ligand/enzyme/enzyme). By iterating over all enzymes in this database, we can extract enzyme-compound and enzyme-coding gene relations using the web service, where the enzymes are provided as Enzyme Commission numbers, compounds are provided as KEGG compound instantiations and genes are denoted by their KEGG gene identifiers. Multiple relations between genes and their enzymes’ metabolites can thus be inferred and indexed using UMLS identifiers, provided with the translation indices as discussed above. Relations are not annotated with literature references. Semantic annotations: *reaction*
- MeSH disease-disease family annotations: Source [61]. Diseases and disease families are annotated with their MeSH identifiers, which can directly be translated to UMLS IDs. Relations are not annotated with literature references. Semantic annotations: *ontology*
- MINT protein-protein interactions: Source [62]. Interactions are annotated by their interacting genes’ UniProt or RefSeq identifiers, which can directly be translated to UMLS IDs. Relations are annotated with PubMed literature references. Semantic annotations: *protein interaction*
- miR2Disease microRNA-disease associations: Source [63]. Diseases are annotated with Disease Ontology identifiers, which can directly be translated to UMLS IDs. miRNAs are annotated with their miRBase identifiers, which are supplemented to the list of concepts. Relations are annotated with article titles and publication years, which have not been translated and incorporated into the databases, since the automated translation of these literature references does not allow for a straightforward computational identification of the database’s literature references. Semantic annotations: *disease miRNA*
- miR2Disease microRNA-gene targeting: Source [64]. Genes are annotated with their HUGO names, which can directly be translated to UMLS IDs. miRNAs are annotated with their miRBase identifiers, which are supplemented to the list of concepts. Relations are annotated with article titles and publication years, which have not been translated and incorporated into the databases, since the automated translation of these literature references does not allow for a straightforward computational identification of the database’s literature references. Semantic annotations: *miRNA target*
- NetworKIN kinase-substrate annotations: Source [65]. Kinases and substrates are annotated with their HUGO gene names, which can directly be translated to UMLS IDs. Relations are not annotated with literature references. Semantic annotations: *phosphorylation*
- OMIM morbid map disease-gene associations: Source [66]. Diseases and genes are provided as their OMIM identifiers, which can directly be translated to UMLS identifiers. References to the literature are available through textual OMIM entries. Relations are annotated to reference this support. Semantic annotations: *disease gene*
- OMIM disease-disease relations: Source [67]. An OMIM disease is said to be related to another disease if a synonym of the OMIM disease contains a substring (separated by “,”, “and”, “of” or “with”, with “type” and numbers removed) that is the name or synonym of this other disease in UMLS. For example, *Charcot-Marie-Tooth, type 4C* is subsequently related to *Charcot-Marie-Tooth*. The originating OMIM identifier can be directly translated to an UMLS identifier and is, as a result of this extraction, related to an UMLS identifier. Semantic annotations: *disease substring*
- TarBase miRNA-gene targeting: Source [68]. Genes are annotated with their HUGO names, which can directly be translated to UMLS IDs. miRNAs are annotated with their miRBase identifiers, which are supplemented to the list of concepts. Relations are annotated with PubMed literature references. Semantic annotations: *miRNA target*
  1. Quality control of the integrated databases

In order to check the quality of the integrated databases, we have repeated the Endeavour benchmark for sets of the databases, by leaving out each database from the data integration and assessing the database’s influence on the benchmark. This assessment was performed on the data as integrated in May 2010, with an AUC of 91.31% if all databases are integrated. Additional Table 3 provides AUCs of the integrated network for each of the databases that were left out for this assessment.

- If a database is left out and the benchmarking results increase significantly, this means that the database has a negative effect on the ranking algorithm, presumably due to the low quality of relations in this database. No databases have been found to have a significantly negative effect on the benchmark where some databases have a slightly negative effect on the performance. Since none of these is significant and since the integrated knowledge from these databases is (subjectively) found to be helpful in the construction of functional hypotheses, these databases have not been removed from the integration.
- If the impact of leaving out a database is significantly decreasing the AUC for the Endeavour test, this database is crucial for the predictive capabilities of BioGraph. CTD gene-disease associations, Gene Ontology Annotations and Medical Subject Headings have been found to have a significantly negative impact on the benchmark, and are consequently considered to be crucial for the scores reported in the manuscript.
  1. Detecting hub nodes by computing a priori probabilities with random walks

A proven technique to detect hubs in a small-world network is to analyze stochastic random walks on the network. Determination of the steady state behavior or limit behavior of performing an infinite stochastic random walk on a network provides a method that measures the relative importance of nodes in a network and ranks authoritative nodes by computing the probability of ending up in a node when performing an infinite random walk on the network.

The algorithm for determining hubs is related to Google’s PageRank^[10]^, used to detect prominent web sites in Internet searches. The algorithm is based upon the notion of a network crawler that visits concepts in the network while randomly following the links between them and thus more frequently visiting authoritative concepts or *hubs*. Hubs are important but generic concepts, unspecific for the discovery of related concepts in biomedical contexts, such as diseases, pathways, etc. Since high ranking hubs are detrimental to our search for specific concepts with respect to a context, we adopt a concept’s *a priori* probability to penalize its ranking with respect to its *a posteriori* relatedness to a contextual concept.

In our setting, the stochastic algorithm for determining the prior probabilities of visiting concepts during an infinite random is applied to measure the relative importance or connectedness of the nodes in the integrated network of biomedical concepts, where the probability of randomly visiting a node is bigger for highly connected nodes, thus detecting hubs as highly probable visited nodes. The computation of a node’s prior probability is straightforward; (1) The network is represented as a probability transition matrix with no damping (i.e., Google’s original implementation of PageRank adopts a damping factor 0.85, we adopt a damping factor 1). (2) The importance or ranking score of a node *i*, i.e., the steady state probability of visiting a node *i*, is given by the *i*th element of the transition matrix’ steady state limit distribution or its unique eigenvector with eigenvalue 1, provided that the transition matrix defines an ergodic Markov chain, which is guaranteed if the network is irreducible and aperiodic (this has been verified for the BioGraph integrated knowledge base). We have adopted the Power Method, optimized for the inherently sparse transition matrix, to compute an approximation of this long term or steady state probability distribution over the concepts. The construction of the integrated network’s transmission matrix is slightly different from PageRank in that the original algorithm assumes a directed graph, where our network consists of essentially undirected relations. We consider each undirected relation in the integrated network as two symmetrical bidirectional edges in order to perform the network analysis algorithm to compute the prior score of the concepts.

More specifically, we represent the graph with *n* concepts as its Markov chain denoted by an *n* x *n* modified adjacency matrix *M’*. For its construction, we first consider the adjacency matrix *M* of the graph, where an element *M_i,j_* is 1 if there’s a relation between concepts *i* and *j* and 0 otherwise. Note that since the relations in the integrated network are bidirectional, *M_i,j_* equals *M_j,i_* for all *i, j.* To obtain the stochastic transition matrix *M’*, we divide each element by its column sum to guarantee all column sums are 1. *M’* now represents the transition probability matrix of the stochastic random walk on the graph where *M’_i,j_* represents the probability to visit state or concept *i* in the next step if the current state of the random walk is *j*. Since the matrix representing the random walk is irreducible and aperiodic and thus ergodic, the limit distribution of the random walk is the unique dominating eigenvector **m^*^** with eigenvalue 1 of the modified adjacency matrix *M’* with

where **m** is any initial probability vector over the states of the Markov chain, following the Perron-Frobenius theorem. The Power Method^[48]^ computes an approximation of the limit distribution, by selecting an initial vector **m** (in our case, this initial vector is the uniform distribution of probabilities over all states or concepts of the graph) and repeatedly multiplying this vector by the modified adjacency matrix *M’* until it has sufficiently converged. In our case, we consider the vector to have converged if the maximal difference between two consecutive vectors of the algorithm no longer decreases. Consequently, the algorithm stops when the algorithm hits the precision of floating point operations on doubles, in this case adopting the IEEE-754 double implementation of Mac OS X.

The resulting vector **m^*^** now provides a numerically sufficient approximation of the probabilities that a random walk visits each node *i* with probability *m^*^_i_*, also designated the ranking score, steady state probability or the *prior accessibility* of the concept in the network. Top ranking concepts in the integrated network are shown in Additional Table 1. The top ranking concept in the network is the Gene Ontology term “Protein binding,” with a probability 0.007, indicating that this concept is visited about once in every 135 steps of a stochastic random walk on the integrated network.

- 1. Computing a posteriori probabilities and ranking relations

In similarity to the algorithm for computing prior probabilities, we can compute the *a posteriori* probabilities to visit any concept with respect to a source concept or source node by performing random walks on the network. Instead of using an infinite random walk, as above, we randomly restart the random walk in the source concept, according to a damping factor, such that the probability of visiting a node nearby the source is expected to be higher than the probability of visiting a node further away from the source node. With a damping factor set at 0.75, the random walk is restarted in the source concept with a probability of 0.25 at each step taken in the random walk. In similarity with the above algorithm for determining prior probabilities, we can describe the random walk with restarts in the source concept as a probability transition matrix and compute its steady state behavior distribution with the Power method.

More specifically, we define the adjacency matrix *M* as above. For the modified version *M’’* that represents the Markov chain of the random walk with restarts, we calibrate each column to represent probabilities with column sums 1 and multiply each element by the damping factor *D*. Finally, we add (1 - *D*) to each element on the *s*th row where *s* denotes the index of the source concept, such that the column sums of the matrix are again 1. The resulting matrix *M’* now represents the transition probability matrix of the Markov chain that represents the random walk with restarts in concept *s,* whose limit behavior can be computed using the Power Method to determine the limit distribution of the stochastic model. As a result, the *a posteriori* probability distribution assigns the highest probabilities to the nodes that are most accessible from the source concept.

A ranking based on this *a posteriori* probability distribution would assign a high ranking to target concepts close to the source concept in the network topology. As discussed before, the integrated network has small world properties and highly unspecific hub would rank high for any source concept because these hubs are highly accessible from any source concept. In order to rank concepts specific to the source concept *s*, we compute the score of each *i*th concept as the ratio of its *a posteriori* probability and square root of its *a priori* probability, with

We adopt this ratio as the ranking score for concepts in relation to the source concept *s*. With this score, concepts are ranked by their vicinity to the source concept, but hub concepts are penalized by their prior accessibility in the global network.­

- 1. Backtracking heuristic for the automated generation of functional hypotheses

The set of *k* shortest or most probable paths that connect a source and target concept is adopted as a set of functional hypotheses to support highly connected source and target concepts. The set can be determined heuristically with a backtracking algorithm, starting from the target concept to find highly probable paths leading toward the source concept, as depicted in Figure 2. The proposed heuristic is based on estimating probabilities to traverse the graph adopting the pre-computed posterior probabilities of each concept in the network with respect to the source concept.

Assume a source concept *s* and a target concept *t* in the integrated network. Let a simple path between *s* and *t* be defined as an ordered list

*P* = { *s, intermediate_1_, …, intermediate_n_, t* }

where each concept in the list is unique and there exists a relation in the network for each of the consecutive nodes in the path, i.e., ( *s, intermediate_1_* ), ( *intermediate_1_, intermediate_2_* ), …, ( *intermediate_n_, t* ) are all edges of the network. The probability of a random walker to traverse this path, provided it starts in *s* and ends in *t* equals

1 / #neighbors( *s* ) Π*_i_*_=1 …_ *_n_* #neighbors( *intermediate_i_* )

where #neighbors( *i* ) denotes the number of neighbors of node *i* in the network.

In order to find highly probable paths leading from *s* to *t*, we find the most accessible neighbor concepts, starting backwards from *t* with respect to *s*, moving toward *s*. At each iteration of the heuristic, we expand the set of neighbors backward to the source concept and prune the set of generated partial paths — with respect to the probability of following this path in a random walk — to a workable number (sufficiently larger than *k*). Eventually, this set of highly accessible paths traces back to the source concept, thus generating a set of highly likely functional paths grounding the indirect relation between the source and target concepts.

More specifically, consider a partial path *P’* in the backtracking algorithm with some intermediate concepts and the target concepts *t*, as follows

*P’* = { *intermediate_1_*, *intermediate_2_*, …, *t* }.

We can estimate the probability to follow this partial path in a random walk from *s* to *t* by considering the posterior probability to arrive at the first intermediate concept of this path with respect to the source concept *s*, and by computing the probability for a random walk to follow the path under construction from this first intermediate concept onward to reach the target *t*, with

Pr( *P’* ) ~ posterior( *intermediate_1_, s*) / Π*_i_*_=1..#intermediates_ #neighbors( *intermediate_i_* ). (1)

The algorithm for backtracking paths, to find *k* paths from *s* to *t* by backtracking the paths from target *t* and and by pruning this set to *K* (>>*k*) paths at each iteration, is provided in the following pseudo code:

*S* = { { *t* } } // initial set of paths contains target

**Repeat**

*S’* = {}

**For each** path *P* = { *a, b, …, t* } in *S*

**If** *a* == *s* // path reached *s*, do not extend

*S’ += P*

**Else** // extend path toward neighbors

**For each** neighbor *n* of *a*

**If** *n* is not in *P* // avoid cycles

*S’* += { *n, a, b, …, t* }

**End**

**End**

**End**

**End**

*S* = Prune *S’* to *K* most likely paths // adopt probability estimate as in (1)

**Until** at least *k* paths in *S* start in *s*

Return top *k* paths in *S* // probability can be exactly computed

At each iteration of the algorithm, we start with a set *S* of partial paths, extend this set of paths by prepending neighbors to each one of the paths and prune this new set such that only the most likely paths according to the estimation as in (1) remain. Initially, *S* contains one partial path, which only has the target concept, i.e., *S =*{ { *t* } }. A each iteration we replace each path { *a*, *b*, …, *t* } in the set of partial paths by a new set of partial paths, i.e., for each neighbor *n* of *a*, we add a new path { *n*, *a*, *b*, …, *t* } to the set of partial paths. Note that we do not extend a path to a neighbor if the path already contains the neighbor, to avoid cycling and in order to end up with simple paths. Paths that have found their way to the source concept are no longer extended, but remain in the set and remain putative candidates for pruning. At the end of each iteration, we prune the new set of paths such that its *K* most probable paths, according to the estimate as in (1), remain. We keep extending likely partial paths to their neighbors and pruning the set of paths to *K* paths until a sufficient number *k* of paths have been found that connect *s* and *t*. We consider these paths as highly likely paths (which is a heuristic but deterministic approximation of the set of most probable paths to traverse the graph from *s* to *t*).

For the generation of hypotheses, we request *k* =10 paths and we prune the set to the *K* = 100 most probable of its partial paths at each iteration. In our implementation, we only extend each of the paths to their *K* most likely neighbors, to save on computation and pruning time. On the full network, the computation of these highly likely paths for the random walk to traverse from *s* to *t* can be computed in no more than a few seconds.

- - 1. Additional Results
  1. Disease-gene prioritization benchmark

All of the diseases and their causal genes have been represented as UMLS concepts to test their relatedness with our prioritization platform.

- - 1. Additional Tables

| **Rank** | **Concept** | **Type** | **UMLS ID** | **Probability** |
| --- | --- | --- | --- | --- |
| 1 | Protein Binding | Function | C0033618 | 0.00739 |
| 2 | Cell Nucleus | Component | C0007610 | 0.00559 |
| 3 | Cytoplasm | Component | C0010834 | 0.00527 |
| 4 | integral to membrane | Component | C1167322 | 0.00522 |
| 5 | Membrane | Component | C0596901 | 0.00471 |
| 6 | Cell membrane | Component | C0007603 | 0.00322 |
| 7 | Metal Ion Binding | Function | C1323274 | 0.00305 |
| 8 | zinc ion binding | Function | C1148621 | 0.00235 |
| 9 | Extracellular | Component | C0521119 | 0.00233 |
| 10 | ATP binding | Function | C1148923 | 0.00231 |
| 11 | Protoplasm | Component | C0175996 | 0.00230 |
| 12 | DNA Binding | Function | C1148673 | 0.00220 |
| 13 | Signal Transduction | Function | C0037083 | 0.00217 |
| 14 | nucleotide binding | Function | C1148916 | 0.00216 |
| 15 | Selenium | Compound | C0036581 | 0.00156 |
| 16 | Vitamin E | Compound | C0042874 | 0.00150 |
| 17 | Regulation of transcription, DNA-dependent | Function | C1158791 | 0.00146 |
| 18 | receptor activity | Function | C1152633 | 0.00146 |
| 19 | Cytoplasmic matrix | Component | C1383501 | 0.00137 |
| 20 | G-protein coupled receptor protein signaling pathway | Pathway | C1155412 | 0.00131 |
| 21 | Fluorouracil | Compound | C0016360 | 0.00131 |
| 22 | Tretinoin | Compound | C0040845 | 0.00130 |
| 23 | tert-Butylhydroperoxide | Compound | C0076150 | 0.00129 |
| 24 | Oxidation-Reduction | Function | C0030012 | 0.00127 |
| 25 | transferase activity | Function | C1151895 | 0.00126 |
| 26 | Selenite | Compound | C0036580 | 0.00124 |
| 27 | transcription factor activity | Function | C1148759 | 0.00121 |
| 28 | Estradiol | Compound | C0014912 | 0.00116 |
| 29 | Mitochondria | Component | C0026237 | 0.00114 |
| 30 | Transcriptional Regulation | Function | C1158770 | 0.00114 |
| 31 | integral to plasma membrane | Component | C1167220 | 0.00114 |
| 32 | Hydrolase activity | Function | C1149632 | 0.00106 |
| 33 | Adenosine Triphosphate | Compound | C0001480 | 0.00102 |
| 34 | multicellular organismal development | Function | C1817464 | 0.00102 |
| 35 | Endoplasmic Reticulum | Component | C0014239 | 0.00095 |
| 36 | Water | Compound | C0043047 | 0.00092 |
| 37 | Progesterone | Compound | C0033308 | 0.00091 |
| 38 | TP53 gene | Gene | C0079419 | 0.00090 |
| 39 | Calcitriol | Compound | C0006674 | 0.00089 |
| 40 | Golgi Apparatus | Component | C0018042 | 0.00085 |
| 41 | decitabine | Compound | C0049065 | 0.00084 |
| 42 | RNA Binding | Function | C1148846 | 0.00082 |
| 43 | Calcium Valproate | Compound | C0006724 | 0.00082 |
| 44 | Biological Transport | Function | C0005528 | 0.00081 |
| 45 | Hydralazine | Compound | C0020223 | 0.00079 |
| 46 | Doxorubicin | Compound | C0013089 | 0.00079 |
| 47 | GRB2 gene | Gene | C1333707 | 0.00078 |
| 48 | calcium ion binding | Function | C1148580 | 0.00076 |
| 49 | Extracellular Space | Other | C0015352 | 0.00076 |
| 50 | Adenosine Diphosphate | Compound | C0001459 | 0.00075 |

1. Additional Table 1: Top 50 hubs or highest ranking concepts of the computation of the *a priori* rank score in the integrated network. The probability denotes the chance to arrive in a concept at any given time when randomly walking on the integrated network. The top concept, *Protein binding*, is visited approximately once every 135 steps of a random walk on the network. For the prioritization of concepts with respect to a source concept, these hubs are penalized as they are less specific for identifying source-specific targets.

| **Disease** | **Genes tested** | **UMLS ID** | **AUC** |
| --- | --- | --- | --- |
| Alzheimer's Disease | 7 | C0002395 | 0.963175 |
| Amyotrophic Lateral Sclerosis | 4 | C0002736 | 0.856323 |
| Anemia | 43 | C0002871 | 0.886218 |
| Anemia, Hemolytic | 13 | C0002878 | 0.96217 |
| Breast Carcinoma | 23 | C0678222 | 0.943615 |
| Carcinoma of the Large Intestine | 21 | C0009402 | 0.955415 |
| Cardiomyopathies | 22 | C0878544 | 0.985262 |
| Cataract | 20 | C0086543 | 0.948638 |
| Charcot-Marie-Tooth Disease | 14 | C0007959 | 0.899821 |
| Deafness | 41 | C0011053 | 0.888322 |
| Diabetes | 26 | C0011847 | 0.914281 |
| Dystonia | 5 | C0013421 | 0.878357 |
| Ehlers-Danlos Syndrome | 10 | C0013720 | 0.915361 |
| Epilepsy | 15 | C0014544 | 0.953232 |
| Ichthyoses | 9 | C0020757 | 0.973799 |
| Leukemia | 109 | C0023418 | 0.861285 |
| Lymphoma | 30 | C0024299 | 0.793848 |
| Mental Retardation | 24 | C0025362 | 0.851548 |
| Muscular Dystrophy | 24 | C0026850 | 0.931996 |
| Myopathy | 41 | C0026848 | 0.919949 |
| Neuropathy | 18 | C0442874 | 0.872345 |
| Obesity | 13 | C0028754 | 0.958196 |
| Parkinson Disease | 9 | C0030567 | 0.959322 |
| Retinitis Pigmentosa | 30 | C0035334 | 0.984988 |
| Spastic Paraplegia | 7 | C0037772 | 0.917061 |
| Spinocerebellar Ataxias | 7 | C0087012 | 0.982104 |
| Usher Syndromes | 7 | C0271097 | 0.994581 |
| Xeroderma Pigmentosum | 10 | C0043346 | 0.997845 |
| Zellweger Syndrome | 9 | C0043459 | 0.998842 |
| Mean |  |  | 92.92% |
| Standard deviation |  |  | 5.20% |

Additional Table 2: Area Under the Receiver Operator Characteristic (ROC) Curve (AUC) for the prioritization of disease genes in the Endeavour benchmark.

| **Left out database** | **AUC (March 2010)** |
| --- | --- |
|  | **Baseline: 91.31** |
| BioGRID | 91.42 |
| CTD compound-gene | 91.43 |
| CTD compound-disease | 91.44 |
| CTD gene-disease | **85.44** |
| DIP | 91.29 |
| GOA | **84.80** |
| HPRD | 91.38 |
| IntAct | 91.43 |
| InterPro domains | 91.29 |
| InterPro ontology | 91.31 |
| InterPro GO annotations | 91.31 |
| KEGG pathways | 91.12 |
| KEGG metabolites | 91.22 |
| MeSH | **89.25** |
| MINT | 91.35 |
| miR2Disease miRNA-target | 91.39 |
| miR2Disease miRNA-disease | 91.53 |
| NetworKIN | 91.33 |
| OMIM | 91.44 |
| TarBase | 91.33 |

Additional Table 3: Effect on the Endeavour benchmark after leaving out each separate database from the data integration process.

1. It should be noted that not all integrated concepts from the originating databases are available as UMLS concepts, even though the UMLS Metathesaurus provides translations between the concept type and its IDs. In the case a concept type is translated using the UMLS Metathesaurus, but specific concepts are not found in the UMLS data, then these concepts and their constituent relations are ignored from integration. [↑](#footnote-ref-2)
